# A Case of Young Brugada Syndrome Patient With Implantable Cardioverter Defibrillator Complication Requiring Device Extraction and Reimplantation

**DOI:** 10.7759/cureus.43576

**Published:** 2023-08-16

**Authors:** Zahid Khan

**Affiliations:** 1 Acute Medicine, Mid and South Essex NHS Foundation Trust, Southend on Sea, GBR; 2 Cardiology, Bart’s Heart Centre, London, GBR; 3 Cardiology and General Medicine, Barking, Havering and Redbridge University Hospitals NHS Trust, London, GBR; 4 Cardiology, Royal Free Hospital, London, GBR

**Keywords:** impedance threshold device, sudden cardiac death (scd), cardiac sudden death, prevention of syncope, vasovagal syncope (vvs), brugada ekg pattern, icd lead, implantable cardioverter-defibrillator (icd), subcut icd, transvenous implantable cardioverter defibrillator

## Abstract

Brugada syndrome is an arrhythmogenic condition characterized by ST-segment elevation and J-point elevation in at least two precordial leads. Most ST segment elevations are associated with myocardial infarction, although other conditions such as pericarditis, channelopathies, and a few genetic conditions should be considered. Brugada syndrome is an inherited cardiac condition associated with an increased risk of sudden cardiac death (SCD). The most common presentation is palpitations or syncopal events in patients presenting to the emergency department. We present the case of a young 26-year-old patient who was diagnosed with Brugada syndrome at the age of 11 following a syncopal event at school and had a transvenous implantable cardioverter defibrillator (ICD) implanted. He was found to have a high lead impedance following a collapse at his routine outpatient device clinic appointment and was transferred to our hospital. He underwent successful transvenous ICD and lead extraction and had a subcutaneous ICD implanted.

## Introduction

Brugada syndrome is an autosomal dominant condition first described in 1992 and characterized by ST-segment elevation and J-point elevation in the right precordial leads V1-V3 and an ST-segment elevation of at least 2 mm [1,2]. It accounts for about 4% of sudden and unexpected cardiac deaths [3]. Brugada and Brugada described eight patients in 1992 with ST-segment elevation in the electrocardiograms (ECGs) that were not described by electrolyte abnormalities or ischemia [2]. Most patients with Brugada syndrome have structurally normal hearts and a male predominance [3]. Patients most commonly present in the third decade, although uncommonly, it can present in children and elderly patients [3]. The most common presentation to the emergency department with the condition is due to palpitations or sudden-onset syncope [4]. Most patients have a benign pattern of the disease, although the one-year mortality rates vary from 18% to 33% in patients presenting with cardiac syncope [4]. It is considered to be the commonest cause of sudden cardiac death (SCD) in patients younger than 50 years old [5].

A review of 104 symptomatic patients with classical Brugada ECG demonstrated that 76 and 28 patients presented with ventricular fibrillation (VF) and syncope, respectively [6]. In a study based on 30 patients, 13 patients presented with syncopal events, and of the remaining 17 patients, 14 had a preceding history of syncopal events [7].

The Brugada syndrome has three types based on ECG findings. Type 1 Brugada syndrome has a coved-shaped ST-segment elevation, a gradually descending ST segment, and negative T waves in at least two right precordial leads V1-V3. Type 2 Brugada syndrome, on the other hand, has saddleback ST-segment elevation with at least 2 mm J-point elevation and biphasic or positive T waves. Type 3 has either a coved or saddleback pattern with at least 1 mm ST-segment elevation, 2 mm J-point elevation, and positive T waves [8]. The latter two types of Brugada patterns are not uncommon in healthy subjects but lack diagnostic specificity [8]. Brugada et al. reported that asymptomatic individuals were at higher risk of sudden death due to the cumulative incidence of ventricular fibrillation, and cardiac arrest occurred in 60% of patients within one year of diagnosis. This is the basis for current guidelines and the use of implantable loop recorders and implantable cardioverter defibrillators (ICDs). We present the case of a young patient who was diagnosed with Brugada syndrome as a child, had an ICD implanted, and later developed ICD-related complications.

## Case presentation

A 26-year-old male Japanese patient was transferred to our hospital for an ICD malfunction. He attended his local hospital in London for a routine device check, where he collapsed during the right ventricular lead testing. He became pale, started jerking, and lost consciousness for about one minute without requiring any shock therapy. ICD interrogation showed that atrial amplitude decreased from 2 mV to 1 mV and right ventricle (RV) sense amplitude decreased from 12 mV to 3 mV from August 2022 to November 2022. The atrial threshold increased from 1.0 V at 0.5 ms in May 2022 to > 4 V at 0.5 ms in October 2022 on capture control and has been out of range on the lead trend since. The device did not show any evidence of ventricular or atrial arrhythmia during this time. His past medical history was significant for a syncopal event as a child when he was 11 years old at school when he was standing for quite some time during a routine sports day. He had an electrocardiogram, cardiac magnetic resonance imaging, and genetic testing during the admission. He was diagnosed with Brugada syndrome and had an ICD implanted to prevent sudden cardiac death (SCD). He had an ICD box changed about a year ago at the referring hospital without any immediate complications. He was not on any regular medications. His mom died suddenly at the age of 34 and was diagnosed with arrhythmogenic right ventricular cardiomyopathy (ARVC) on post-mortem; his 23-year-old sister also has ARVC and is doing fine; and his maternal uncle died suddenly from a cardiac condition, but the patient could not recall the exact nature of the disease. He lives with his spouse, is a lifelong non-smoker, and has occasional drinks over the weekend.

**Figure 1:**
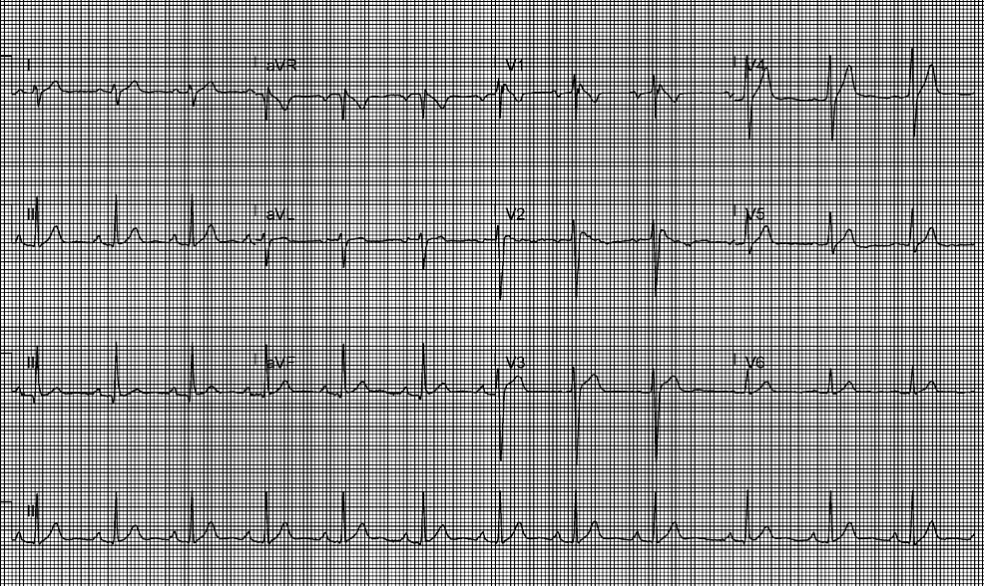
Electrocardiography shows type 1 Brugada pattern with ST segment elevation in leads V1-V3

He was transferred as an emergency on the direct arrhythmia admission pathway to our cardiac electrophysiology unit. On arrival, he was hemodynamically stable; his HR was 77 bpm, his BP was 127/66 mmHg, his RR was 20 breaths per minute, his SpO_2_ was 100% on room air, and the temperature was 37.3 °C. His laboratory tests were all normal. ECG showed ST segment elevation in right precordial leads V1-V3 and a normal QT interval (Figure 1). Echocardiography showed normal biventricular size and function with no regional wall motion abnormalities (Videos 1-2). ICD interrogation showed a DDDR (dual-pacing, dual-sensing, dual-response, rate-adaptive) setting, a lower rate limit of 40 bpm, and a VF therapy zone of 200 bpm. The RA impedance was stable at 390 Ohms, thresholding at 3 V, gradually trending up from <1 V in May 2022 and 2 V in August 2022. The RV lead showed a stable pace impedance of 450 Ohms, a shock impedance (coil > can) of 55 Ohms, and a stable R wave of 2.4-2.9 mV, rapidly trending down from >12 mV in August 2022 and 3-6 mV in November 2022. The RV threshold showed intermittent capture at 7.5 V at 1.5 ms, a marked increase from 2.5 V at 0.5 ms in August 2022 and 1.5 V at 0.5 ms in May 2022. The patient was discussed in the device multidisciplinary meeting (MDT), and the consensus was to offer device and lead extraction followed by subcutaneous ICD (S-ICD) implantation. The patient gave written consent to the proposed plan. He had a blood group and save prior to the procedure, which showed blood group B, positive Rh D, and an Rh phenotype reported as probably R1R2 and negative K antigen. As a result, the patient required a special type of blood transfusion arrangement in case he required blood transfusion during or after the device extraction. This, along with the lack of general anesthesia slot availability, led to his procedure cancellation, and the patient self-discharged from the hospital against medical advice.

**Video 1:** Parasternal long axis view shows normal left ventricular ejection fraction.

**Video 2:** Apical 4 chamber view shows normal left ventricular ejection fraction.

**Figure 2:**
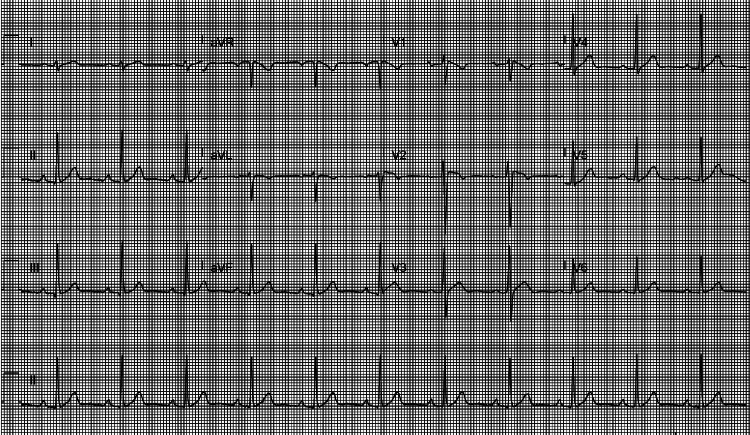
Electrocardiography shows normal sinus rhythm with mild ST elevation in leads V1-V3

**Figure 3:**
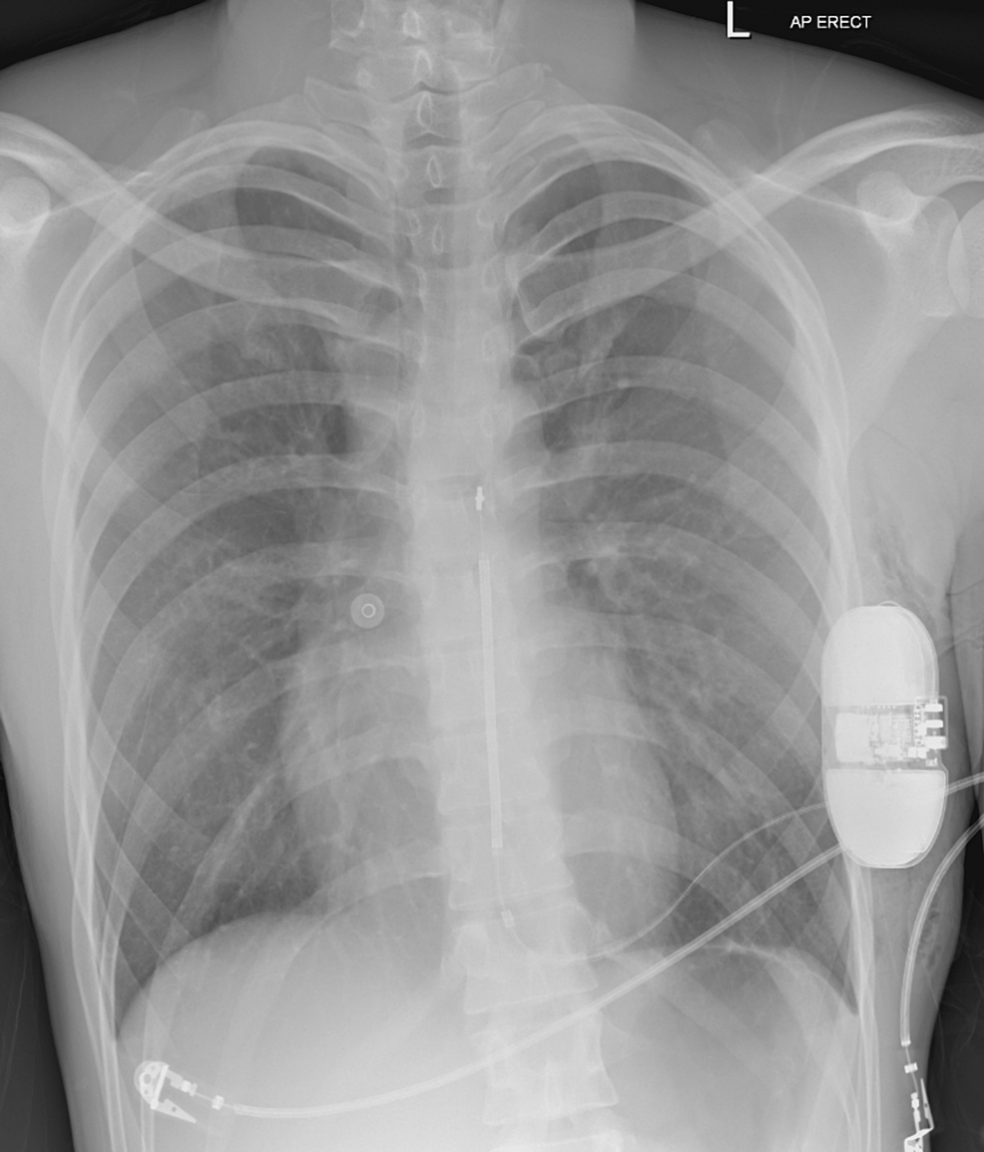
Chest radiography showing satisfactory subcutaneous implantable cardioverter defibrillator leads position

He was scheduled for outpatient device extraction, which he underwent in July this year, followed by S-ICD implantation. S-ICD was successful in all three leads (Table 1). A repeat ECG showed normal sinus rhythm, and chest radiography showed proper lead position (Figures 2-3). The device check was satisfactory, and the patient was discharged home on analgesia with outpatient follow-up in the device and arrhythmia clinic. He has remained stable since, with no further complications.

**Table 1:** S-ICD screening S-ICD: subcutaneous implantable cardioverter defibrillator

Sternal lead position	Sternal lead summary							
Lead	Supine	Standing/sitting	Original implant location	Implant post standing	Stretch	Optional posture	Morphology consistent between postures?	Mark all acceptable leads
Primary lead - III	Ok	Ok	Ok	Ok	Ok	-	Yes ✓ No	✓
Secondary lead - II	Ok	Ok	Ok	Ok	Ok	-	Yes ✓ No	✓
Alternate lead - I	Ok	Ok	Ok	Ok	Ok	-	Yes ✓ No	✓

## Discussion

Brugada syndrome detection has improved since its first introduction in 1992, and more patients with the disease are getting diagnosed now. Brugada syndrome has three distinct patterns described earlier, with type 1 being the more classical pattern of the disease [3]. The pathophysiology remains somewhat controversial between the repolarization and depolarization hypotheses, but according to Wilde et al., the evidence is more in favor of the former [9]. Most evidence from clinical studies suggests a right ventricular conduction delay to be the likely pathophysiology behind Brugada syndrome [2,9]. It is more prevalent in young adults of both genders of Southeast Asian descent and accounts for 0.05-0.06% of cases of SCD in patients of all ethnic groups [4]. It is inherited as an autosomal dominant condition with variable penetrance that results from sodium channel dysfunction due to three distinct mutations on the cardiac sodium channel gene SCN5A [5,10]. Patients who experience sudden syncopal events, nocturnal agonal respiration, ventricular fibrillation, ventricular tachycardia, and a family history of sudden cardiac death [5]. The diagnostic challenges include patients having seizure-like episodes and misinterpretation of the ECG for possible ST-elevated myocardial infarction (STEMI) [5]. Both clinical and ECG criteria are valuable to help in the diagnosis of Brugada syndrome, and the provocative tests with class 1 antiarrhythmic sodium channel blocker drugs like ajmaline, flecainide, or procainamide can unmask the diagnosis by converting the subtle saddleback ST-segment elevation in types 2 and 3 patterns into more typical type 1 coved- pattern ST-segment elevation, which helps to confirm the diagnosis [6].

The role of the programmed electrical stimulation (PES) test is used in risk stratifying patients, although concerns have been raised about its prognostic value. A study on PES demonstrated its positive predictive value to be 50% and its negative predictive value to be 46%, and this study concluded that PES should not be relied on as the only method to risk stratify patients at risk of SCD. The decision to implant a transvenous or S-ICD should be weighed against the quality of life and the risk of inappropriate shock that could result from abnormal repolarization or T-wave oversensing [11].

A few studies have been performed on ICD-related complications in patients with Brugada syndrome [12,13]. El-Battrawy et al. reported that the most common ICD complication was appropriate and inappropriate shocks (18.5% and 18.1%, respectively), followed by lead failure and fracture at 5.4% [12]. Other ICD complications include lead perforation (0.7%), lead dislodgement (1.7%), infection (3.9%), subclavian vein thrombosis (0.3%), endocarditis (0.1%), and psychiatric problems (1.5%). Most inappropriate shocks were attributed to supraventricular arrhythmias (13.7%), noise (3.7%), and T-wave oversensing (2.5%). Another study reported a complication rate of 28%, and the main complications in the first month included pneumothorax, pericardial effusion, lead displacement, and venous thrombosis, including pulmonary embolism and hematoma [13]. Late complications included lead failure or infection requiring extraction and reimplantation of the leads and generator, deeper implantation of the leads, device failure, inappropriate shocks that were due to lead dysfunction, T-wave oversensing, sinus tachycardia, and atrial arrhythmia [13]. Inappropriate shocks were managed through pulmonary vein isolation, sotalol, or hydroquinidine in most patients [12].

Another study on ICD implantation in children and adolescents with Brugada syndrome reported device-related complications in 14% of patients, which included a fracture of the lead, a lead dislocation, and pulse generator migration in 60%, 20%, and 20% patients, respectively [14]. This study also demonstrated a reduced incidence of inappropriate shocks in patients who had ICD implanted after 2010 (10%) compared to those who had ICD implanted before 2010 (33%). Similarly, the percentage of patients presenting with an ICD-related complication was 10% and 26% in patients who had ICDs implanted after and before 2010, respectively. Our patient had an ICD implanted before 2010 and had generator changes a year ago. He did not have any inappropriate shocks; however, given his young age and the fact that he never received a shock, he was offered a S-ICD, which was implanted as an outpatient without any complications.

## Conclusions

In conclusion, Brugada syndrome is an autosomal dominant inherited condition with a strong risk of sudden arrhythmogenic cardiac death. Patients with Brugada syndrome are mostly young and die from sudden cardiac death. There is usually a family history of sudden cardiac death, and an electrocardiogram and clinical examination are key to the diagnosis. Most patients require an implantable cardioverter defibrillator to avoid sudden cardiac death, and the preference of the type of device between a transvenous and a subcutaneous ICD should be discussed with the patient. ICD therapy is an important preventative strategy for symptomatic young patients with a high risk of sudden cardiac death. The patient's risk of sudden cardiac death needs to be assessed independently of programmed electrical stimulation.
